# International Nursing doctoral education: quality matters

**DOI:** 10.1590/1518-8345.0000.4149

**Published:** 2024-11-04

**Authors:** Shaké Ketefian, Silvina Malvarez

**Affiliations:** ^1^ University of Michigan, School of Nursing, Ann Arbor, Michigan, United States of America.; ^2^ University of Cordoba, School of Public Health, National, Cordoba, Argentina.

**Figure:**
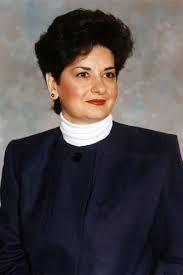

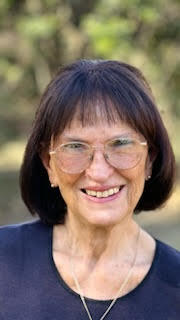

Nursing doctoral education has expanded rapidly worldwide. An informal anecdotal count suggests that there are 39 countries offering doctoral education, for a tentative total of 657 programs (conducted by the authors). Yet, before we celebrate this highly positive development we need to assure ourselves that these programs have mechanisms in place to ascertain that their offerings are indeed of high quality. [It is important to note that there are several countries that are known to have doctoral programs but could not be included in our count as we could not obtain an estimated number of programs they offered].

It is likely that each country, and indeed each institution, has its own procedures for ascertaining quality. These may be processes for evaluating individual courses, programs or at the level of the institution. In this manner, most schools tend to have some activities related to quality assurance. In most instances these institutional level mechanisms would typically be built around guidance provided at the governmental or professional society level.

Yet, it is important that there be some level of coherence, and understanding of what the goals of schools are in offering research-focused doctoral education. There is a tendency by individual schools to develop their programs in isolation, whereby each determines its own educational objectives and sets forth to evaluate them. Yet country-level efforts provide a greater degree of assurance that the overall societal level health care needs are considered in articulating the goals of doctoral education, so that student attainments are articulated, along with the stated goals through which programs aim to help their students attain competence that are responsive to health care needs of their national populations.

Evaluations can occur at the individual course level by asking students and alumni to assess the quality of their experiences, and of course the extent to which course faculty have assisted students in meeting course objectives. Students and alumni are in a good position to also assess the extent to which they are developing skill sets relevant to the research and other activities in which they are or will be engaged. Same applies to faculties’ assessment of their own courses and of the extent to which students attain the specified skills at the end of each course.

Some countries have established their own national standards; these can be used to develop broad or specific criteria to: design program elements, and to obtain information from relevant parties as mentioned, that should include faculties, administrators, as well as students and alumni of inputs.

 Over two decades ago under an initiative of the newly formed International Network for Doctoral Education in Nursing (INDEN) ^(^ 1 ^)^ , whereby a team developed criteria for assessing the quality of international research-focused nursing doctoral programs; these were widely promulgated. Subsequently, several individuals who had participated in the effort collaborated as an expanded group and developed an instrument that operationalized the criteria that had been developed under INDEN sponsorship. This effort took 8 to 10 years and resulted in an instrument that can be used for evaluation of international research-focused doctoral programs ^(^ 2 ^)^ . The instrument is in the form of a scale, and taps several areas deemed important; the domains being addressed are program, faculty, resource, and evaluation domains, in addition to a section where relevant information is sought on the respondents, who were faculty members and students (locating alumni proved too complex). The instrument was tested in several countries and they each presented their own national findings in their home journals. The countries where data collection occurred were: Australia, Canada, several European countries, Japan, South Africa, Thailand, the United Kingdom, and the United States; the instrument reported ^(^ 2 ^)^ incorporates findings from the worldwide sample. 

In most of the Latin American countries governmental bodies establish standards for doctoral education, as they do for all university-level offerings. This task is performed by special national commissions that establish accreditation criteria to ensure quality.

It is noteworthy that expression of statements of philosophy and guidance are useful guides, but ultimately, these ideals need to be converted into the form of valid and reliable instruments or tests, through which objective information can be obtained from relevant populations so that such information can be used for various purposes to ensure quality.

 In a similar spirit, as an example of how countries might develop their own documents via their professional societies to guide the educational and training process for their member schools, we mention the work of the American Association of Colleges of Nursing (AACN), that has developed a document, which is periodically revised, that presents a philosophy of doctoral education, along with suggested content areas and approaches to enable graduates to provide optimum service to their respective societies ^(^ 3 ^)^ . 

The rapid expansion of international doctoral education suggests that a large number of these programs may be new; it is therefore a good time for programs to begin giving thought to how they might build in evaluation techniques and strategies at the program level, course level and indeed, at the national level through collaboration with colleagues.
