# Supplementary material for: International Nursing doctoral education: quality matters
Source: Rev Lat Am Enfermagem. 2024 Nov 4;32:e4149. doi: 10.1590/1518-8345.0000.4149 (PMC11537261; doi:10.1590/1518-8345.0000.4149)
Supplement: Translation

# Translation: La educación doctoral en Enfermería internacional: la calidad importa

**DOI:** 10.1590/1518-8345.0000.4148

**Published:** 2024-10-17

**Authors:** Shaké Ketefian, Silvina Malvarez

**Affiliations:** ^1^ University of Michigan, School of Nursing, Ann Arbor, Michigan, Estados Unidos de América.; ^2^ University of Cordoba, School of Public Health, National, Cordoba, Argentina.

La educación doctoral en enfermería se ha expandido rápidamente en todo el mundo. Un recuento informal anecdótico sugiere que hay 39 países que ofrecen formación de doctorado, lo que suma un total tentativo de 657 programas (realizado por los autores). Sin embargo, antes de celebrar este avance altamente positivo, debemos asegurarnos de que estos programas cuentan con mecanismos para verificar que sus ofertas son realmente de alta calidad. [Es importante mencionar que hay varios países que se sabe que tienen programas de doctorado, sin embargo, no fueron incluidos en nuestro conteo porque no pudimos obtener una estimación del número de programas que ofrecían].

Es probable que cada país, y de hecho cada institución, tenga sus propios procedimientos para comprobar la calidad. Estos pueden ser procesos de evaluación de cursos individuales, programas, o a nivel institucional. De esta manera, la mayoría de las escuelas tienden a tener algunas actividades relacionadas con el aseguramiento de la calidad. En la mayoría de los casos, estos mecanismos a nivel institucional se construirían en torno a la orientación proporcionada a nivel gubernamental o de la sociedad profesional.

Sin embargo, es importante que haya cierto nivel de coherencia y comprensión de cuáles son los objetivos de las escuelas al ofrecer educación doctoral centrada en la investigación. Existe una tendencia por parte de cada escuela a desarrollar sus programas de forma aislada, con lo cual cada una determina sus propios objetivos educativos y se propone evaluarlos. Sin embargo, los esfuerzos a nivel de país brindan un mayor grado de garantía de que las necesidades generales de atención de salud a nivel social se tienen en cuenta al articular los objetivos de la educación doctoral, de modo que los logros de los estudiantes se articulen junto con los objetivos establecidos por medio de los cuales los programas buscan ayudar a sus estudiantes a adquirir competencias que respondan a las necesidades de cuidados de salud de sus poblaciones nacionales.

Las evaluaciones pueden realizarse a nivel de curso individual, solicitando a los estudiantes y ex alumnos que evalúen la calidad de sus experiencias y, por supuesto, hasta qué punto el personal docente del curso los ha ayudado a alcanzar los objetivos del curso. Los estudiantes y ex alumnos también están en una buena posición para evaluar en qué medida están desarrollando conjuntos de habilidades relevantes para la investigación y otras actividades en las que están o estarán involucrados. Lo mismo se aplica a la evaluación que hacen las facultades de sus propios cursos y del grado en que los estudiantes alcanzan las competencias especificadas al final de cada curso.

Algunos países han establecido sus propios estándares nacionales, que pueden utilizarse para desarrollar criterios amplios o específicos para diseñar elementos del programa y obtener información de las partes relevantes mencionadas, que deben incluir profesores, administradores, así como aportes de estudiantes y ex alumnos.

 Hace más de dos décadas, gracias a una iniciativa de la recién formada *International Network for Doctoral Education in Nursing* (INDEN) ^(^ 1 ^)^ , un equipo desarrolló criterios para evaluar la calidad de los programas internacionales de doctorado en enfermería centrados en la investigación; estos criterios se difundieron ampliamente. Posteriormente, varias personas que habían participado en el trabajo colaboraron como un grupo ampliado y desarrollaron un instrumento que ponía en práctica los criterios que se habían desarrollado bajo el patrocinio de INDEN. Este esfuerzo tomó entre 8 y 10 años y dio como resultado un instrumento que puede utilizarse para la evaluación de programas de doctorado internacionales centrados en la investigación ^(^ 2 ^)^ . El instrumento tiene forma de escala y aborda varias áreas consideradas importantes; los dominios abordados son el programático, el del plantel docente, el de los recursos y el de la evaluación, además de un apartado donde se busca información relevante sobre los encuestados, que eran profesores y estudiantes (localizar a los ex alumnos resultó demasiado complejo). El instrumento fue probado en varios países, y cada uno de ellos presentó sus propios hallazgos en sus revistas nacionales. Los países donde se realizó la recolección de datos fueron: Australia, Canadá, varios países europeos, Japón, Sudáfrica, Tailandia, Reino Unido y Estados Unidos; el instrumento reportado ^(^ 2 ^)^ incorpora los hallazgos de la muestra mundial. 

En la mayoría de los países de América Latina, los organismos gubernamentales establecen estándares para la educación doctoral, como lo hacen para toda la oferta de nivel universitario. Esta tarea está a cargo de comisiones nacionales especiales que establecen criterios de acreditación para asegurar la calidad.

Es de destacar que la expresión de enunciados filosóficos y orientaciones son guías útiles, pero, en última instancia, estos ideales deben convertirse en forma de instrumentos o pruebas válidos y confiables, por medio de los cuales se pueda obtener información objetiva de poblaciones relevantes para que dicha información pueda usarse para diversos propósitos para garantizar la calidad.

 De forma similar, como ejemplo de cómo los países podrían desarrollar sus propios documentos por medio de sus sociedades profesionales para guiar el proceso educativo y de formación de sus escuelas miembro, mencionamos el trabajo de la *American Association of Colleges of Nursing* (AACN), que ha desarrollado un documento, que se revisa periódicamente, que presenta una filosofía de la educación doctoral, junto con áreas de contenido y enfoques sugeridos para permitir que los graduados brinden un servicio óptimo a sus respectivas sociedades ^(^ 3 ^)^ . 

La rápida expansión de la educación doctoral internacional sugiere que un gran número de estos programas pueden ser nuevos; por lo tanto, es un buen momento para que los programas comiencen a pensar en cómo podrían incorporar técnicas y estrategias de evaluación a nivel de programa, de curso y, seguramente, a nivel nacional mediante la colaboración con colegas.

# Translation: Educação internacional de doutorado em Enfermagem: a qualidade importa

**DOI:** 10.1590/1518-8345.0000.4150

**Published:** 2024-10-17

**Authors:** Shaké Ketefian, Silvina Malvarez

**Affiliations:** ^1^ University of Michigan, School of Nursing, Ann Arbor, Michigan, Estados Unidos de América.; ^2^ University of Cordoba, School of Public Health, National, Cordoba, Argentina.

A educação de doutorado em Enfermagem expandiu-se rapidamente em todo o mundo. Uma contagem anedótica informal sugere que há 39 países oferecendo educação de doutorado, para um total provisório de 657 programas (realizada pelos autores). Contudo, antes de comemorarmos esse desenvolvimento altamente positivo, precisamos nos certificar de que esses programas tenham mecanismos para garantir que suas ofertas sejam de fato de alta qualidade. [É importante mencionar que há vários países que são conhecidos por terem programas de doutorado, porém, não puderam ser incluídos em nossa contagem porque não conseguimos obter uma estimativa do número de programas que eles ofereciam].

É provável que cada país, e de fato cada instituição, tenha seus próprios procedimentos para verificar a qualidade. Esses podem ser processos para avaliar cursos individuais, programas, ou de nível institucional. Dessa maneira, a maioria das escolas tende a ter algumas atividades relacionadas à garantia de qualidade. Na maior parte dos casos, esses mecanismos de nível institucional normalmente seriam construídos em torno de orientações fornecidas no nível governamental ou de sociedade profissional.

No entanto, é importante que haja algum nível de coerência e compreensão sobre quais são os objetivos das escolas ao oferecer educação de doutorado focada em pesquisa. Há uma tendência de cada escola desenvolver seus programas isoladamente, em que cada uma determina seus próprios objetivos educacionais e se propõe a avaliá-los. Porém, os esforços no âmbito nacional fornecem um maior grau de garantia de que as necessidades gerais de cuidados de saúde a nível social sejam consideradas na articulação dos objetivos da educação de doutorado, de modo que as realizações dos alunos sejam articuladas, juntamente com as metas declaradas por meio das quais os programas visam ajudar seus alunos a obter competências que sejam responsivas às necessidades de cuidados de suas populações nacionais.

As avaliações podem ocorrer no nível do curso individual, solicitando que alunos e ex-alunos avaliem a qualidade de suas experiências e, claro, até que ponto o corpo docente do curso os ajudou a atingir os objetivos do curso. Os alunos e ex-alunos estão em boa posição para também avaliar até que ponto estão desenvolvendo conjuntos de habilidades relevantes para a pesquisa e outras atividades em que estão ou estarão envolvidos. O mesmo se aplica à avaliação das faculdades sobre os seus próprios cursos e à extensão em que os alunos atingem as competências especificadas no final de cada curso.

Alguns países estabeleceram seus próprios padrões nacionais; eles podem ser usados para desenvolver critérios amplos ou específicos para elaborar elementos do programa e obter informações de partes relevantes, conforme mencionado, que devem incluir professores, administradores, bem como contribuições de alunos e ex-alunos.

 Há mais de duas décadas, por iniciativa da recém-formada *International Network for Doctoral Education in Nursing* (INDEN) ^(^ 1 ^)^ , uma equipe desenvolveu critérios para avaliar a qualidade dos programas de doutorado em enfermagem com foco em pesquisa internacional; estes foram amplamente divulgados. Posteriormente, vários indivíduos que participaram do trabalho colaboraram como um grupo expandido e desenvolveram um instrumento que operacionalizou os critérios que foram desenvolvidos sob o patrocínio do INDEN. Esse esforço levou de 8 a 10 anos e resultou em um instrumento que pode ser usado para a avaliação de programas de doutorado internacionais focados em pesquisa ^(^ 2 ^)^ . O instrumento tem a forma de uma escala e aborda diversas áreas consideradas importantes; os domínios abordados são programa, corpo docente, recursos e avaliação, além de uma seção onde são buscadas informações relevantes sobre os entrevistados, que eram membros do corpo docente e alunos (localizar ex-alunos era muito complexo). O instrumento foi testado em vários países, e cada um apresentou suas próprias descobertas em seus periódicos nacionais. Os países onde ocorreu a coleta de dados foram: Austrália, Canadá, vários países europeus, Japão, África do Sul, Tailândia, Reino Unido e Estados Unidos; o instrumento relatado ^(^ 2 ^)^ incorpora descobertas da amostra mundial. 

Na maioria dos países latino-americanos, órgãos governamentais estabelecem padrões para a educação de doutorado, assim como para todas as ofertas de nível universitário. Essa tarefa é realizada por comissões nacionais especiais que determinam critérios de acreditação para garantir a qualidade.

Vale ressaltar que a expressão de declarações de filosofia e orientação são guias úteis, mas, em última análise, esses ideais precisam ser convertidos na forma de instrumentos ou testes válidos e confiáveis, por meio dos quais informações objetivas possam ser obtidas de populações relevantes, para que tais informações sejam usadas para vários propósitos, a fim de garantir a qualidade.

 De forma similar, como um exemplo de como os países podem desenvolver seus próprios documentos por meio de suas sociedades profissionais para orientar o processo educacional e de formação para suas escolas associadas, mencionamos o trabalho da *American Association of Colleges of Nursing* (AACN), que desenvolveu um documento, revisado periodicamente, que apresenta uma filosofia de educação de doutorado, juntamente com áreas de conteúdo e abordagens sugeridas para permitir que os graduados forneçam o melhor serviço às suas respectivas sociedades ^(^ 3 ^)^ . 

A rápida expansão da educação internacional de doutorado sugere que um grande número desses programas pode ser novo; portanto, é um bom momento para os programas começarem a pensar em como podem desenvolver técnicas e estratégias de avaliação em nível de programa, de curso e, certamente, em nível nacional por meio da colaboração com colegas.
